# Mizuo‐Nakamura phenomenon in an Indian male

**DOI:** 10.1002/ccr3.1990

**Published:** 2019-01-13

**Authors:** Rohit Agarwal, Koushik Tripathy, Gopal Bandyopadhyay, Koushik Basu

**Affiliations:** ^1^ Department of Ophthalmology ASG Eye Hospital Lake Town, Kolkata India

**Keywords:** congenital stationary night blindness, Oguchi disease, X‐linked cone dystrophy, X‐linked retinoschisis

## Abstract

The authors present a 20‐year‐old myopic male who showed golden color of fundus (Mizuo‐Nakamura phenomenon) in light and normal color after long dark adaptation. This phenomenon is associated with an abnormally slow dark adaptation and is typically noted in Oguchi disease, a variant of congenital stationary night blindness.

A 20‐year‐old myopic (−4.5DS bilaterally) man complained of delayed dark adaptation. On indirect ophthalmoscopy, areas of a peculiar sheen of the fundus were noted at the posterior pole (Figures 1B,C and 2B,C). Well defined discolored areas (Figure 3A,B,C) were seen outside the arcade,^1^ which gradually changed color to shiny golden areas after exposure to bright light or repeated flashes of fundus camera (Mizuo‐Nakamura phenomenon [MNP],^2^ Figures 1B,C, 2B,C, and 3D). After 6 hours of dark adaptation, the color of the fundus came back to near normal (Figures 1A and 2A).

**Figure 1:**
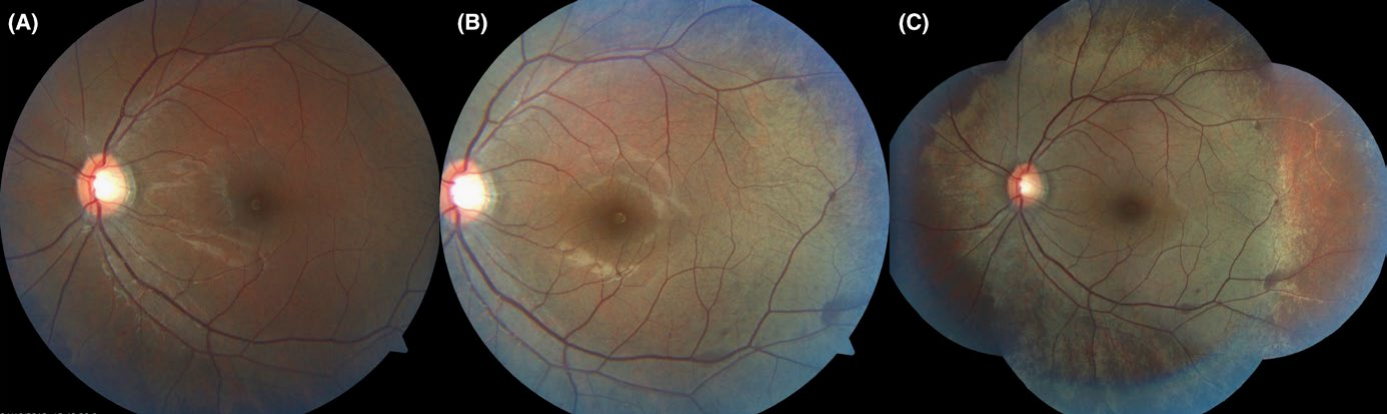
A, The left fundus achieved near normal color after long dark adaptation. B, Immediately after exposure to flash of fundus camera, the golden color was evident. C, The montage image shows golden discoloration and peripheral granular appearance

**Figure 2:**
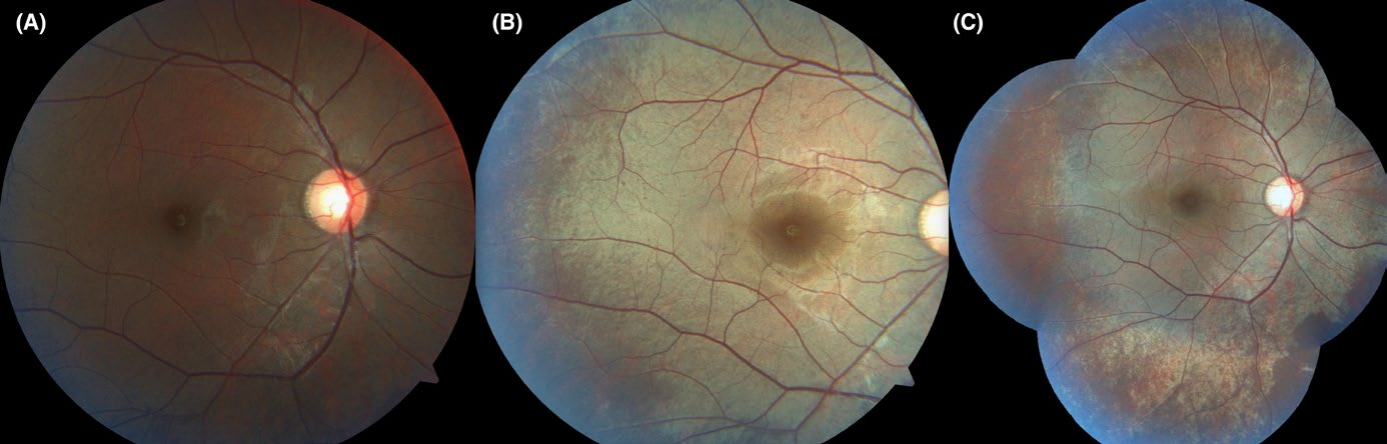
A, The right fundus looks normal in color after dark adaptation of 6 h. B, The golden color of fundus was noted after light exposure. C, Shows a montage image revealing the prominence of retinal vessels

**Figure 3:**
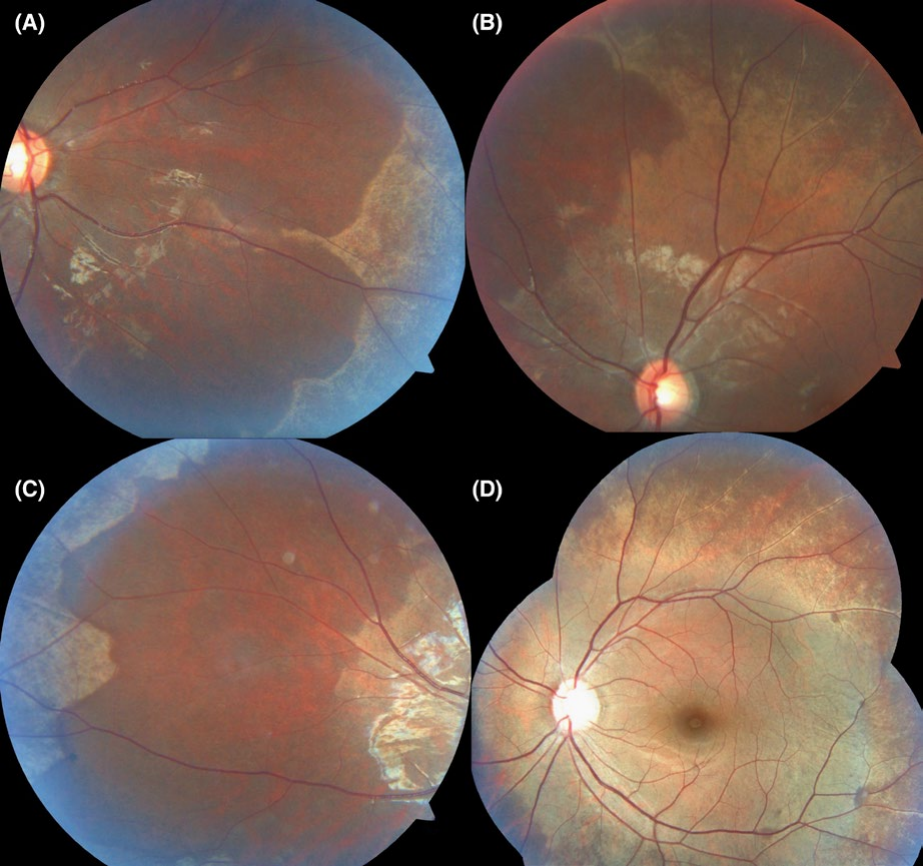
A, B, C, Shows the typical mid‐peripheral areas of retinal discoloration. D, The golden shine was accentuated when the brightness of the fundus camera‐flash was increased

Mizuo‐Nakamura phenomenon has been reported in various diseases including Oguchi disease (OD, a form of congenital stationary night blindness), X‐linked retinoschisis, and X‐linked cone dystrophy.^1^ Oguchi disease is an autosomal recessive disorder usually caused by mutation of arrestin or rhodopsin kinase. These molecules stop the phototransduction cascade, and their mutation leaves the rhodopsin molecules in photoactivated state for long duration. On electroretinogram, cone responses are normal.^2^ Mixed rod‐cone response has a negative configuration. Rod response in normal test condition shows absent a‐ and b‐waves, which appear normal after long dark adaptation in the initial single flash, but rod response again becomes extinguished with repeated flashes.^2^

## CONFLICT OF INTEREST

None declared.

## AUTHOR CONTRIBUTION

RA and KT: had full access to all data of the manuscript and take responsibility for the integrity of the data. GB, KT, RA, and KB: were responsible for acquisition of data. All the authors were involved in manuscript concept and design, analysis and interpretation of data, drafting of the manuscript, and critical revision of the manuscript for important intellectual content.
